# Development of Woody Plants

**DOI:** 10.3390/plants15070988

**Published:** 2026-03-24

**Authors:** Min Chen, Yi-Shu Wang, Hong-Xiang Peng, Jin-Zhi Zhang

**Affiliations:** National Key Laboratory for Germplasm Innovation & Utilization of Horticultural Crops, College of Horticulture and Forestry Science, Huazhong Agricultural University, Wuhan 430070, China; minchen970402@webmail.hzau.edu.cn (M.C.); wang2917790919@163.com (Y.-S.W.); 19863834331@163.com (H.-X.P.)

Research on woody plant development is a highly integrated interdisciplinary field that systematically investigates trees across various scales, from microscopic to macroscopic levels. It encompasses not only the morphogenesis and growth patterns of plant structures (e.g., vegetative and reproductive organs) but also the underlying physiological processes (e.g., photosynthesis and stress responses) and their genetic regulation. Furthermore, it examines how environmental factors like light, temperature, and soil affect development and shape adaptive strategies. Expanding on this basis, investigations into reproductive strategies, wood formation, and wood quality offer critical knowledge for understanding tree life cycles and enabling their economic use. Modern research actively employs biotechnology and genetic engineering to enhance desirable traits, while increasingly focusing on the adaptability and resilience of woody plants in the context of climate change, aiming to provide a scientific basis for sustainable forest management and ecosystem conservation. Given the field’s significance and the systematic advances made in recent years, a comprehensive synthesis of its current progress is both timely and warranted. Such a synthesis will also serve as a valuable foundation for inspiring future research in woody plant development.

The morphological development of woody plants spans from seed germination and the formation of root, stem, and leaf organs to age-related increases in plant height, stem thickening, and canopy complexity. Research progress on the genetic improvement of woody tree architecture is currently slow due to their inherent biological complexity, specifically their complex structure, long growth cycle, and extended juvenile phase. Thus, research efforts are now centered on systematically identifying the functional genes governing key architectural traits and deciphering their molecular regulatory networks. The shoot apical meristem (SAM), which is established during embryogenesis, represents the primordial source of all aerial plant parts. The *KNOX* (*KNOTTED1-like homeobox*) gene family is key to SAM maintenance and functional specification. Class I *KNOX* genes, such as *STM* (*SHOOTMERISTEMLESS*), *KNAT1*/*BP* (*BREVIPEDICELLUS*), *KNAT2*, and *KNAT6*, are critical for SAM activity, balancing stem cell renewal with organ formation [1]. *STM*, for example, is required for SAM initiation in embryos; its loss results in seedlings lacking a functional SAM and producing only cotyledons [2]. Studying the architectural traits of woody plants is essential for understanding their growth patterns, optimizing silvicultural management, improving productivity, and ultimately cultivating high-quality timber [3,4]. An update article analyzed the architectural characteristics of five olive cultivars (*Chemlali*, *Chetoui*, *Koroneiki*, *Meski*, and *Picholine*) under semi-arid climate conditions in Tunisia, providing insights for sustainable olive crop management [5]. The authors demonstrated significant differences among cultivars in traits such as branch angle, shoot length, and inflorescence distribution. Understanding the consistency of these architectural features allows researchers to utilize them in future studies to harmonize growth patterns. This approach can reduce management complexity and enhance the efficiency of mechanized operations such as pruning and harvesting. Additionally, as complex biological structures, the stability of woody plants in urban environments is essential for both survival and public safety. In this regard, a recent study investigated the mechanical properties, stress distribution, and performance of tree fork structures, offering valuable insights for safety assessment and pruning strategies in urban tree management [6]. Future research could further integrate computer simulation with real-time monitoring technology to develop a structural safety early-warning model for trees, thereby offering systematic solutions for risk management and intelligent oversight in urban forestry.

The development of woody plants includes vegetative and reproductive growth stages. Reproductive development involves a series of intricate processes: flower bud differentiation, flowering and pollination, fertilization and fruit set, as well as seed maturation. During these stages, developmental defects such as flower bud abortion, low pollen viability, poor fruit set, or inferior seed quality often occur due to nutrient deficiency, environmental stress (e.g., drought, low temperature), or hormonal imbalance. Therefore, artificial interventions—such as optimized fertilization, exogenous hormone application, pruning, or environmental control—can directly improve these processes [7,8]. By addressing developmental defects, optimizing resource allocation, and enhancing stress tolerance, such measures play a crucial role in stabilizing yield, improving quality, and accelerating breeding progress. In this context, a recently updated study has investigated the effects of exogenous phytohormones and boron fertilizer on reproductive development, fruit set, and yield in the macadamia cultivar ‘A4’ (*Macadamia integrifolia*, *M. tetraphylla*) [9]. The authors recommended an integrated management strategy: foliar spray of 0.02% boron at the floral bud stage, followed by 2 mg/L 6-BA at full bloom, and a combined application of 0.02% boron and 0.2 mL/L brassinosteroid at early fruit set. This strategy effectively increased yield while reducing fruit abscission, offering a practical approach for production optimization. Further exploration of such exogenous regulation patterns in early-stage breeding screening and trait evaluation will help accelerate the fixation of desirable traits and enhance the efficiency of variety improvement. In addition, molecular approaches have revolutionized the study of woody plant reproduction. RNA-seq analyses in *Populus* have delineated temporal gene expression waves during reproductive bud development, identifying chromatin remodelers like *DRNL* (*DORNROSCHEN-like*) as key upstream regulators of tissue-specific morphogenesis [10]. Integrative multi-omics strategies, combining transcriptomics, metabolomics, and hormone profiling, have uncovered dynamic shifts in gibberellin (GA) and jasmonate levels during floral transition in *Lagerstroemia*, linking phytohormone gradients to internode elongation and SAM proliferation [11]. Future directions include single-cell RNA-seq to resolve spatiotemporal heterogeneity in reproductive tissues and synthetic biology approaches to engineer hormone-responsive promoters for precision breeding. By bridging classical physiology with cutting-edge omics, research on woody plant reproduction not only advances our fundamental understanding of the field but also catalyzes innovations in forestry and horticulture. Climate change has profoundly altered the developmental processes of woody plants. Rising temperatures disrupt phenology, extending the growing season while increasing the risk of late frosts; drought stress inhibits photosynthesis and growth, leading to tree decline or mortality; and frequent extreme-weather events cause direct physical damage. Concurrently, indirect effects such as increased pest and disease pressure and reduced reproductive success accumulate, ultimately driving shifts in forest distribution and community structure [12,13]. In this context, enhancing forest resilience through adaptive management has become a critical challenge. Emerging technologies such as cold plasma (CP) treatment are being explored to improve disease resistance, seedling growth, seed germination, and overall crop performance [14,15,16,17]. A recently updated study has revealed that the effects of CP seed treatment on antioxidants, sugars, and pigments in Norway spruce (*Picea abies* (L.) H. Karst.) needles are genotype-dependent [18]. By examining changes in photosynthetic pigments, phenolic compounds, soluble sugars, and lipid peroxidation over a two-year CP treatment period, the authors aimed to better understand CP’s potential in enhancing plant resilience to environmental stress. Uncovering such genotype-specific responses is therefore important for developing advanced seed treatment strategies to improve forestry productivity and forest health. Future research should evaluate the potential impacts of emerging technologies on long-term tree growth, wood quality, ecosystem functions, and soil microbial communities to ensure the ecological safety and sustainability of these technologies.

Many woody species have evolved diverse regulatory mechanisms to adapt to endogenous factors and complex environmental conditions. Advancements in multi-omics technologies have unveiled the transcriptional networks underpinning these adaptations. For example, *Populus* under drought conditions exhibit coordinated upregulation of *AP2*/*ERF*, *MYB*, and *NAC* transcription factors, which regulate osmotic homeostasis and oxidative stress responses [19]. However, challenges remain, including the need to validate candidate genes under fluctuating climatic conditions and the need to address organ-specific responses in woody plants. Future research integrating omics technologies, structural biology, and computational modeling will pave the way for sustainable solutions to global environmental challenges. In natural environments, light plays a crucial role in woody plant development [20]. Under shaded conditions, most plants exhibit a series of adaptive morphological and physiological changes, known as the shade-avoidance response [21]. When shading reduces leaf photosynthesis, plants strategically adjust carbon allocation by reducing resource investment in roots and stems, thereby enhancing overall growth rate and adaptability to low-light environments [22,23]. Studies have reported that decapitation can reduce inter-leaf shading and improve light penetration and utilization within the canopy, leading to higher yields [20,24]. A recent study confirmed the effectiveness of decapitation in annual *Pinus yunnanensis* and showed that the main stem maintained isometric growth across all shading treatments, accounting for 26.8% of individual plant biomass while exhibiting dominant biomass allocation and high shade sensitivity [25]. These findings indicate that decapitation (artificial pruning) enhances light-use efficiency and yield by mitigating leaf shading and optimizing canopy light exposure. Therefore, this study not only provides theoretical insights into regulating woody plant development but also offers a scientific basis for pruning management in agricultural and forestry production. In Mediterranean ecosystems, intense and prolonged droughts threaten the growth and reproduction of young tree seedlings [26]. Another study evaluated the early growth and short-term physiological adaptation of seven sclerophyllous forest species in central Chile to water restriction and shading [27]. The authors found that all species responded similarly to these treatments, with changes in specific leaf area, stomatal conductance, chlorophyll content, photosynthetic rate, and intrinsic water-use efficiency, indicating convergent adaptive patterns to shade and drought. To address these issues, further research is needed to explore the interactive effects of multiple environmental factors, particularly the mechanism by which frequent compound stresses under climate change affect seedling establishment and forest regeneration.

Research on woody plant development holds great significance for plant genetic improvement, sustainable forest resource utilization, and ecosystem responses to climate change. Continued investigation in this field remains essential to support future breeding programs and forest management practices. This series of update articles elucidates recent advances in key areas of woody plant development, including morphogenesis, growth patterns, reproductive regulation, and climate adaptation. We anticipate future scientific breakthroughs based on the insights gathered in this Special Issue, as well as progress in developing eco-friendly and innovative strategies to enhance the adaptability and resilience of forest ecosystems under global climate change. Moving forward, efforts should focus on strengthening the integration of multidisciplinary approaches, technological innovation, and ecological practices to achieve predictable management of tree development processes and to improve the adaptability of both plantation and urban forests. Furthermore, it is imperative to establish an ecological safety assessment framework to ensure that the application of emerging technologies balances long-term production benefits with ecosystem sustainability.
